# Standard Errors of Kernel Equating: Accounting for Bandwidth Estimation

**DOI:** 10.1177/01466216211066601

**Published:** 2022-03-07

**Authors:** Kseniia Marcq, Björn Andersson

**Affiliations:** 1 205777 University of Oslo, Oslo, Norway

**Keywords:** achievement testing, classical test theory, equating, item response theory, standard errors

## Abstract

In standardized testing, equating is used to ensure comparability of test scores across multiple test administrations. One equipercentile observed-score equating method is kernel equating, where an essential step is to obtain continuous approximations to the discrete score distributions by applying a kernel with a smoothing bandwidth parameter. When estimating the bandwidth, additional variability is introduced which is currently not accounted for when calculating the standard errors of equating. This poses a threat to the accuracy of the standard errors of equating. In this study, the asymptotic variance of the bandwidth parameter estimator is derived and a modified method for calculating the standard error of equating that accounts for the bandwidth estimation variability is introduced for the equivalent groups design. A simulation study is used to verify the derivations and confirm the accuracy of the modified method across several sample sizes and test lengths as compared to the existing method and the Monte Carlo standard error of equating estimates. The results show that the modified standard errors of equating are accurate under the considered conditions. Furthermore, the modified and the existing methods produce similar results which suggest that the bandwidth variability impact on the standard error of equating is minimal.

Standardized testing is commonly used for assessing individual achievement and its results greatly influence high-stakes decisions ranging from university admissions to various industry certifications. Standardized testing generally requires alternate test forms to be administered on multiple occasions. As a consequence, the tests often differ in difficulty from one administration to another, which poses a challenge with respect to comparability and fairness of the resulting test scores. In order to address this challenge, a statistical procedure known as equating is employed with the paramount goal of adjusting the scores on the test forms so that they yield interchangeable results (Kolen & Brennan, 2014).

Observed-score equating is one of the fundamental methods used in test equating. Rooted in classical test theory, it is concerned with establishing the equivalence of the observed scores on two test forms and includes both linear and equipercentile equating functions (von Davier, 2011). In this study, we focus on an equipercentile observed-score equating method called kernel equating, which was initially introduced by Holland and Thayer (1989) and further developed by von Davier et al. (2004).

The conceptual framework of kernel equating follows that of equipercentile observed-score equating and posits a series of steps to obtain the equated scores: (1) pre-smoothing of the data to reduce sampling variability, (2) obtaining discrete score probability distributions, (3) obtaining continuous approximations to the discrete score distributions, (4) calculating the equating function, and (5) calculating the standard errors of equating (SEEs) (von Davier, 2011; von Davier et al., 2004). A feature that distinguishes kernel equating from other equipercentile methods is that the continuous approximations of the score probability distributions are achieved through kernels that utilize bandwidth parameters. The bandwidths allow the density functions to be as smooth as possible while retaining the properties of the original distributions. Estimating such parameters, however, introduces additional sampling variability. This variability is typically not accounted for when calculating the standard errors of kernel equating, and therefore constitutes a threat to their accuracy (Holland et al., 1989; von Davier et al., 2004).

Accurate estimation of the SEE is integral to making correct inferences and comparisons. When estimated incorrectly, it can lead to unjustified certainty. One previous study derived modified standard errors of kernel equating when using a variant of the Silverman’s rule of thumb for bandwidth estimation (Andersson et al., 2014). The current study derives modified SEEs when using the more commonly applied approach to estimate the bandwidth by minimizing a penalty function. Such an approach is more generally appropriate and does not rely on a particular distributional assumption for the test scores. Thus, the objective of this article is to introduce a modified method of calculating the SEE which accounts for the additional variability stemming from the bandwidth estimation. The new approach is compared via simulations to the current method of calculating the SEE (Holland et al., 1989) across several sample sizes and test lengths.

We structure this article as follows. In the subsequent section, we give a brief background to the kernel method of equating and expand on the issue of bandwidth estimation and how it influences sampling variability. We also discuss how the standard errors of kernel equating are currently estimated. Next, the asymptotic variance of the bandwidth parameter estimator is derived and is incorporated in a modified method for calculating the SEE. This modified method is further verified and compared to the existing method in a simulation study. Lastly, the results are reported and discussed.

## The Kernel Method of Test Equating

### Data Collection Designs

An observed-score equating procedure consists of two fundamental components, namely, the data collection design and the equating method (von Davier et al., 2004). Hence, before we focus on the equating itself, it is essential to review, if only briefly, the common approaches to collecting the data. There are several data collection designs widely used in practice and they can roughly be divided into two categories: designs which use examinees from a common population taking both test forms and designs which use common items on the test forms (von Davier et al., 2004). The first category of data collection designs includes the equivalent groups, the single group, and the counterbalanced designs, and the second category includes the common-item non-equivalent groups design. In this study, we focus on the equivalent groups design, where two independent random samples are drawn from a common population, *P*, and one group takes test form *X*, while the other takes test form *Y*. In the following, we will use *X* and *Y* to denote both the test forms and the random variable corresponding to the test scores from each of the test forms.

The choice of an appropriate data collection design is subject to considerations like the available sample size, time, and costs. The designs subsequently affect the equating procedure implying that some designs, such as the equivalent groups design, allow for a relatively straightforward comparison between the test forms. Other designs are much more complex, such as the common-item non-equivalent groups design. A more detailed account of the considerations and procedures involved in various data collection designs can be found in von Davier et al. (2004).

### Kernel Equating

In the following, we adopt the notation of von Davier et al. (2004). Let the target population be *T*, the possible score values on the test form *X* be *x*_*j*_ for *j* = 1, …, *J*; and let the possible score values on the test form *Y* be *y*_*k*_ for *k* = 1, …, *K*. Thus, we define the score probabilities as

(1) $$r_{j}=\text{Prob}\left\{X=x_{j}|T\right\},$$

and

(2) $$s_{k}=\text{Prob}\left\{Y=y_{k}|T\right\}.$$

Further, an equipercentile equating function is defined in terms of the cumulative distribution functions (CDFs) which are given by

(3) $$F\left(x\right)=\text{Prob}\left(X\leq x\right)=\sum_{j,x_{j}\leq x}r_{j},$$

and

(4) $$G\left(y\right)=\text{Prob}\left(Y\leq y\right)=\sum_{k,y_{k}\leq y}s_{k}.$$

When the CDFs are continuous, we obtain the equipercentile equating function of *X* to *Y* from

(5) $$y={\text{Equi}}_{\text{Y}}\left(x\right)=G^{-1}\left(F\left(x\right)\right).$$

Strictly speaking, however, most score distributions are discrete, and their continuous approximations are required. Kernel equating addresses this problem by introducing a series of steps which can be applied to various data collection designs and which provides continuous CDFs. The steps of kernel equating are pre-smoothing, estimation of the score probabilities, continuous approximation to the discrete score distributions, equating, and calculating the SEE (von Davier et al., 2004). We now briefly review the first two steps and dedicate subsequent subsections to present the remaining steps in more detail as they pertain to the subject at hand.

#### Pre-Smoothing

In the pre-smoothing step, a parametric statistical model is fitted to the observed data. This can be done by fitting log-linear or item response theory (IRT) models to the data. The methods are described in detail in Andersson and Wiberg (2017), and Holland and Thayer (1987), and are not repeated here.

#### Estimation of the Score Probabilities

Having estimated the score distributions with a pre-smoothing model, the score probabilities can be obtained using a linear or non-linear transformation which, following von Davier et al. (2004), we call the design function. The design function depends on the data collection design. For instance, consider the equivalent groups design and let $r={\left(r_{1},\ldots ,r_{J}\right)}^{t}$ denote the column vector of the score probabilities of *X* and $s={\left(s_{1},\ldots ,s_{K}\right)}^{t}$ denote the column vector of the score probabilities of *Y*. The design function (DF) is then a simple identity function, that is

(6) $$\text{DF}\left(r,s\right)=\left(\begin{array}{cc} I_{J} & 0\\ 0 & I_{K} \end{array}\right)\left(\begin{array}{c} r\\ s \end{array}\right)=\left(\begin{array}{c} r\\ s \end{array}\right),$$

where **I**_*J*_ and **I**_*K*_ are *J* × *J* and *K* × *K* identity matrices. Design functions for other data collection designs are given explicitly in von Davier et al. (2004).

### Continuous Approximation and Equating

The third step in kernel equating, distinguishing it from other equipercentile methods, is how the continuous approximations to the discrete CDFs, $F_{h_{X}}\left(x\right)$ and $G_{h_{Y}}\left(y\right)$ to *F*(*x*) and *G*(*y*), are obtained. In kernel equating, this is achieved by applying a kernel with a smoothing bandwidth parameter (von Davier et al., 2004). There are different kernels available in the literature (Lee et al., 2008), but in this study we focus on the Gaussian kernel, which is most commonly used. Following the notation of von Davier et al. (2004), let Φ(⋅) denote the CDF of the Gaussian distribution, and let *h*_*X*_ denote the bandwidth parameter. Then, the Gaussian kernel smoothing of the distribution of *X* has the CDF defined by

(7) $$F_{h_{X}}\left(x\right)=\sum_{j}r_{j}\Phi \left(R_{j_{X}}\left(x\right)\right),$$

where *R*_*jX*_(*x*) is given by

(8) $$R_{jX}\left(x\right)=\frac{x-a_{X}x_{j}-\left(1-a_{X}\right)\mu_{X}}{a_{X}h_{X}},$$

and *a*_*X*_, *μ*_*X*_, and $\sigma_{X}^{2}$ are functions of ***r***, that is

(9) $$\mu_{X}=\sum_{j}x_{j}r_{j},$$

(10) $$\sigma_{X}^{2}=\sum_{j}{\left(x_{j}-\mu_{X}\right)}^{2}r_{j},$$

(11) $$a_{X}=\sqrt{\frac{\sigma_{X}^{2}}{\sigma_{X}^{2}+h_{X}^{2}}}.$$

${\hat{G}}_{h_{Y}}\left(y\right)$ is defined analogously.

It is evident from equations (7)–(11) that for the continuous approximation to be carried out, the bandwidth parameters *h*_*X*_ and *h*_*Y*_ have to be estimated. The primary goal of introducing such parameters is to make the density functions as smooth as possible while retaining the properties of the original distributions. Various methods of estimating the bandwidth parameter have been suggested in previous research (Andersson et al., 2014; von Davier et al., 2004). Of particular interest to this study is a method described in Holland and Thayer (1989) and von Davier et al. (2004), which estimates the bandwidth parameter by minimizing a penalty function, named PEN_1_ (*h*_*X*_) in von Davier et al. (2004), with respect to the bandwidth. The penalty function itself is based on the squared distances between proportions and the density function and is given by

(12) $$\;{\text{PEN}}_{1}\left(h_{X}\right)=\sum_{j}\left(r_{j}-f_{hX}{\left(x_{j}\right)}^{2}\right),$$

where $f_{h_{X}}\left(x_{j}\right)$ is the density function, found by differentiating Equation 7 with respect to *x*, that is

(13) $$f_{h_{X}}\left(x\right)=\sum_{j}r_{j}\phi \left(R_{jX}\left(x\right)\right)\frac{1}{a_{X}h_{X}},$$

and *R*_*jX*_(*x*) is given in Equation 8. The density obtained as a result of estimating the bandwidth by minimizing PEN_1_ is typically a good approximation of the discrete score distribution. Note that sometimes it can be beneficial to smooth the density function further, in which case an additional component named PEN_2_ in von Davier et al. (2004) can be applied combined with PEN_1_. However, because of the complexity in accounting for estimation variability with PEN_2_, we focus exclusively on PEN_1_ in the present study.

Once the continuous approximations are obtained, the equating function estimator for equating *X* to *Y* is given by

(14) e^Y(x;r^,s^)=G^hY−1(F^hX(x;r^);s^).

The equating function for equating *Y* to *X* is analogous and found by substitution.

### Standard Error of Kernel Equating

The SEE is the measure of random equating error or uncertainty which stems from the equating function being subject to estimation and thereby sampling variability. We largely base this subsection on the work of Holland et al. (1989), who derived the asymptotic standard error for the kernel method of equating using the standard delta method for computing large sample approximations to the sampling variances of functions of statistics. Before proceeding, we see it appropriate to briefly introduce the multivariate delta method (Rao, 1973).

Adopting the notation of Rao (1973), let the (*k* × 1)-dimensional random vector $\sqrt{n}\left(T_{kn}-\theta_{k}\right)$ converge to a multivariate normal distribution with zero mean and covariance Σ, where *T*_*kn*_ is an estimator, *θ*_*k*_ is the true parameter vector, and *n* denotes the sample size. Let *g* denote a vector-valued function with components *g*_1_,…, *g*_*q*_, such that all the entries of *g* are differentiable. Then, $\sqrt{n}\left(g\left(T_{kn}\right)-g\left(\theta_{k}\right)\right)$ converges to a multivariate normal distribution with zero mean and covariance of *G*Σ*G*^′^, that is

(15) $$\sqrt{n}\left(g\left(T_{kn}\right)-g\left(\theta_{k}\right)\right)\overset{d}{\to }N\left(0,G\Sigma G^{\prime }\right),$$

where *G* is the (*q* × *k*) Jacobian matrix of partial derivatives of *g* with respect to *θ*_*k*_. In this study, the equivalent of *T*_*kn*_, *θ*_*k*_, and *g* are the estimator of the score probabilities, the true score probabilities, and the equating function, respectively. The delta method was employed by Holland et al. (1989), where they defined the SEE for equating *X* to *Y* by

(16) SEEY(x)=Var(e^Y(x;r^, s^)).

The SEE for equating *Y* to *X* is defined analogously.

Treating the bandwidth parameters *h*_*X*_ and *h*_*Y*_ as constants, Holland et al. (1989) assert that all the uncertainty in the equating function comes from the estimation of the score probabilities ***r*** and ***s***. Hence, the variance of the equating function, and in turn the SEE, reflects the data collection design, the choice of the pre-smoothing technique used in the estimation of the population score probabilities, and the equating function itself.

Reiterating the notation used previously, let ***r*** and ***s*** define the vectors of the pre-smoothed score distributions. The calculation of the SEE per Holland et al. (1989) then requires two components: the vector $\partial_{e_{Y}}$ with derivatives of the equating function *e*_*Y*_ with respect to ***r*** and ***s***, and the asymptotic covariance matrix Σ(r^,s^) . Using the delta method (Rao, 1973), the variance of the equating function e^Y can then be expressed as

(17) Var(e^Y(x;r^,s^))=∂eYΣ(r^,s^)[∂eY]′,

where ***s*** and Σ(r^,s^) is the covariance matrix of the independently estimated score probabilities, given by

(18) Σ(r^,s^)=[Σr^00Σs^].

The matrix Σ(r^,s^) has dimensions (*J* + *K*) × (*J* + *K*) where *J* is the dimension of ***r*** and *K* is the dimension of ***s*** (von Davier et al., 2004). The calculation of Σ(r^,s^) for different equating designs can be found in Andersson and Wiberg (2017), Holland et al. (1989) and von Davier et al. (2004).

The second component, $\partial_{e_{Y}}$ , can be defined as follows

(19) $$\partial_{e_{Y}}=\left[\frac{\partial e_{Y}}{\partial r},\frac{\partial e_{Y}}{\partial s}\right].$$

Recalling Equation 14, the derivatives needed to compute $\partial_{e_{Y}}$ are defined in Holland et al. (1989) as

(20) $$\frac{\partial e_{Y}}{\partial r_{j}}=\frac{1}{G^{\prime }}\frac{\partial F\left(x,\;r\right)}{\partial r_{j}},$$

(21) $$\frac{\partial e_{Y}}{\partial s_{k}}=-\frac{1}{G^{\prime }}\frac{\partial G\left(e_{Y}\left(x\right);s\right)}{\partial s_{k}},$$

where $\frac{\partial e_{Y}}{\partial r}$ is a row vector with dimensions 1 × *J*, $\frac{\partial e_{Y}}{\partial s}$ is a row vector with dimensions 1 × *K*, and *G*^′^ is the density evaluated at *e*_*Y*_ (*x*), that is

(22) $$G^{\prime }=\frac{\partial G\left(e_{Y}\left(x\right);s\right)}{\partial y},$$

and

(23) $$\frac{\partial F\left(x;r\right)}{\partial r_{j}}=\Phi \left(R_{jX}\left(x;r\right)\right)-M_{jX}\left(x;r\right)\frac{\partial F\left(x;r\right)}{\partial x},$$

where $\frac{\partial F\left(x;r\right)}{\partial x}$ is given in Equation 13, *R*_*jX*_ (*x*; ***r***) in Equation 8, and

(24) $$M_{jX}\left(x;r\right)=\frac{1}{2}\left(x-\mu_{X}\right)\left(1-a_{X}^{2}\right)z_{jX}^{2}+\left(1-a_{X}\right)x_{j},$$

where *z*_*jX*_ is defined as

(25) $$z_{jX}=\frac{x_{j}-\mu_{X}}{\sigma_{X}}.$$

The derivatives of *e*_*X*_ are analogous to those above and can be computed by substitution.

At this point, it is important to emphasize that the method of SEE calculation described above treats the bandwidth parameters *h*_*X*_ and *h*_*Y*_ as fixed and not as functions of ***r*** and ***s***. Hence, the additional variability introduced by the bandwidth estimation is currently not accounted for in the calculation of the SEEs, and consequently poses a challenge with respect to their accuracy (Holland et al., 1989; von Davier et al., 2004).

## Accounting for Bandwidth Estimation Variability in Kernel Equating

In this section, we first derive the bandwidth parameter estimator variance and then introduce a modified method for the calculation of the analytical SEE that accounts for bandwidth estimation variability.

### Asymptotic Variance and Standard Error of the Bandwidth Parameter Estimator

Recalling the standard delta method restated in the previous section (Rao, 1973), it is important to note that the bandwidth parameter estimator is not defined explicitly but rather as an implicit function of other asymptotically normal variables. Therefore, we use a generalization of the delta method presented by Benichou and Gail (1989) which facilitates computing the asymptotic variance of the implicitly defined bandwidth parameter estimator. Following the notation of von Davier et al. (2004), *h*_*X*_ denotes the bandwidth parameter selected to minimize PEN_1_ defined by Equation (12) and ***r*** denotes the vector of estimated score probabilities. Consider further that PEN_1_ is a continuously differentiable function of the estimated score probabilities ***r*** in *h*_*X*_, and the function is minimized so that $\frac{\partial {\text{PEN}}_{1}}{\partial h_{X}}=0$ . Applying the implicit function theorem (Rao, 1973), we can then define *h*_*X*_ as a function of ***r*** such that $h_{X}=g_{h_{X}}\left(r\right)$ , and compute the partial derivatives of $g_{h_{X}}\left(r\right)$ with respect to ***r*** as

(26) $$\frac{\partial g_{h_{X}}\left(r\right)}{\partial r}=-{\left(\frac{\partial^{2}{\text{PEN}}_{1}}{\partial h_{X}^{2}}\right)}^{-1}\frac{\partial^{2}{\text{PEN}}_{1}}{\partial h_{X}\partial r^{\prime }},$$

where $\frac{\partial^{2}{\text{PEN}}_{1}}{\partial h_{X}^{2}}$ is a scalar second order partial derivative of PEN_1_ with respect to *h*_*X*_ and $\frac{\partial^{2}{\text{PEN}}_{1}}{\partial h_{X}\partial r^{\prime }}$ is a 1 × *J* vector of second-order partial derivatives of PEN_1_ with respect to ***r***. The $\frac{\partial^{2}{\text{PEN}}_{1}}{\partial h_{X}^{2}}$ and $\frac{\partial^{2}{\text{PEN}}_{1}}{\partial h_{X}\partial r^{\prime }}$ derivatives are unequivocally calculated using the chain rule and implicit differentiation. The equations, however, are lengthy, and we summarize them in the appendix.

Let Σr^ denote the asymptotic covariance matrix of the estimated score probabilities ***r*** with dimensions *J* × *J* where *J* is the dimension of ***r***. By applying the delta method for implicit functions (Benichou & Gail, 1989), we can define the asymptotic variance of the bandwidth parameter estimator ${\hat{h}}_{X}$ as

(27) Var(h^X)=∂ghX(r)∂rΣr^[∂ghX(r)∂r]′,

and its standard error as

(28) $$\text{SE}\left({\hat{h}}_{X}\right)=\sqrt{\text{Var}\left({\hat{h}}_{X}\right)}.$$

The variance and the standard error of ${\hat{h}}_{Y}$ are analogous to those given for ${\hat{h}}_{X}$ and can be computed by substituting *X* by *Y* and ***r*** by ***s***.

### Standard Error of Equating Accounting for Bandwidth Variability

To account for the bandwidth estimation variability when computing the SEEs, we apply the chain rule together with the delta method and obtain a modified expression for the SEEs (Cox, 1984). Treating ${\hat{h}}_{X}$ and ${\hat{h}}_{Y}$ as functions of the score probability estimators r^ and s^ , we redefine Equation 14 by adding a term which accounts for the bandwidth estimation variability to obtain

(29) Var(e^Y(x;r^,s^,h^X(r^),h^Y(s^)))=∂eY(x)∂(r,s)Σ(r^,s^)[∂eY(x)∂(r,s)]′+∂(hX(r),hY(s))∂(r,s)∂eY(x)∂(hX,hY)×Σ(r^,s^)[∂(hX(r),hY(s))∂(r,s)∂eY(x)∂(hX,hY)]′,

where $\frac{\partial e_{Y}\left(x\right)}{\partial \left(r,s\right)}$ is presented in equations (19)–(25) and Σ(r^,s^) in Equation 18. When evaluating these expressions in practice, the true parameters are replaced by the parameter estimates. The additional components are then a (2 × (*J* + *K*))-matrix of partial derivatives of the bandwidth parameters as functions of the estimated score probabilities with respect to the estimated score probabilities, $\frac{\partial \left(h_{X}\left(r\right),h_{Y}\left(s\right)\right)}{\partial \left(r,s\right)}$ , calculated following Equation (26), that is

(30) $$\frac{\partial \left(h_{X}\left(r\right),h_{Y}\left(s\right)\right)}{\partial \left(r,s\right)}=\left[\begin{array}{cc} \left(-{\left(\frac{\partial^{2}{\text{PEN}}_{1}}{\partial h_{X}^{2}}\right)}^{-1}\frac{\partial^{2}{\text{PEN}}_{1}}{\partial h_{X}\partial r^{\prime }}\right) & 0\\ 0 & \left(-{\left(\frac{\partial^{2}{\text{PEN}}_{1}}{\partial h_{Y}^{2}}\right)}^{-1}\frac{\partial^{2}{\text{PEN}}_{1}}{\partial h_{Y}\partial s^{\prime }}\right) \end{array}\right],$$

and $\frac{\partial e_{Y}\left(x\right)}{\partial \left(h_{X},h_{Y}\right)}$ , a (2 × *J*)-matrix of first-order derivatives of the equating function with respect to the bandwidth parameters, *h*_*X*_ and *h*_*Y*_, defined by

(31) $$\frac{\partial e_{Y}}{\partial h_{X}}=\frac{1}{G^{\prime }}\sum_{j}r_{j}\frac{\partial \Phi \left(R_{jX}\left(x\right)\right)}{\partial h_{X}},$$

where

(32) $$\frac{\partial \Phi \left(R_{jX}\left(x\right)\right)}{\partial h_{X}}=\sum_{j}r_{j}\phi \left(R_{jX}\left(x\right)\right)\frac{\partial R_{jX}\left(x\right)}{\partial h_{X}},$$

and

(33) $$\frac{\partial e_{Y}}{\partial h_{Y}}=-\frac{1}{G^{\prime }}\sum_{k}r_{k}\frac{\partial \Phi \left(R_{kY}\left(y\right)\right)}{\partial h_{Y}},$$

where

(34) $$\frac{\partial \Phi \left(R_{kY}\left(y\right)\right)}{\partial h_{Y}}=\sum_{k}r_{k}\phi \left(R_{kY}\left(y\right)\right)\frac{\partial R_{kY}\left(y\right)}{\partial h_{Y}},$$

and *G*^′^ is defined in Equation 22, with *R*_*jX*_(*x*) and *R*_*kY*_(*y*) given in Equation 8. Lastly, we define the SEE which accounts for bandwidth variability by

(35) SEEY(x)=Var(e^Y(x;r^,s^,h^X(r^),h^Y(s^))).

## Simulation Study

To confirm the accuracy of the presented derivations, we conducted a simulation study to evaluate the estimators of the standard error of the bandwidth parameter estimator and the modified SEEs. We evaluated the estimated standard errors with respect to the Monte Carlo standard errors and compared the modified standard errors to the original standard errors that do not account for the bandwidth estimation.

### Simulation Design

Data for two test forms *X* and *Y* were simulated using the two-parameter logistic (2-PL) model within the framework of IRT (de Ayala, 2009), where test lengths of 20, 40, and 80 items were considered. The discrimination parameters for both test forms were selected from the *U* (0.5, 2)-distribution and the difficulty parameters for one test form were selected from the *N* (0.25, 1)-distribution and the other from the *N* (−0.25, 1)-distribution. These distributions were considered to mimic realistic item parameters found in standardized testing (National Center for Education Statistics, 2004).

**Figure 1.**
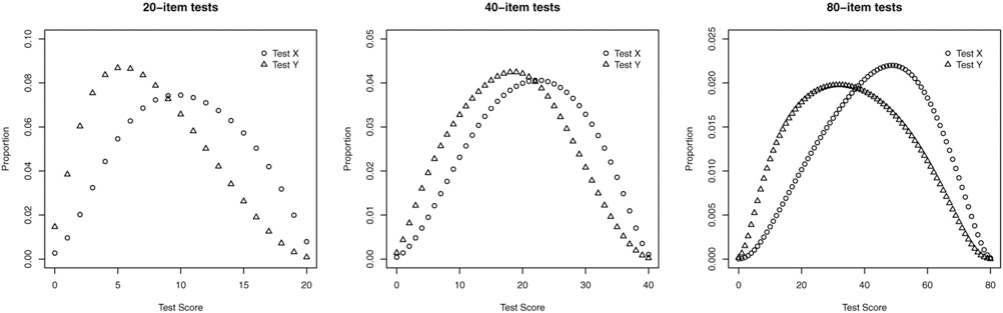
Score distributions with 20, 40, and 80 items, for each test *X* and *Y*.

The equivalent groups design was used in which two independent random samples of individuals are drawn from a single common population and where each random sample takes either of the test forms *X* and *Y* (von Davier et al., 2004). Dictated by the design, no differences in the latent distributions were present between the groups. The latent distributions were set to the standard normal distribution. The equivalent groups design was considered because of its simplicity. Relative to other data collection designs, it provided an opportunity for direct comparison of the results on the test forms *X* and *Y* without additional considerations or assumptions. The score distributions for the tests *X* and *Y* with 20, 40, and 80 items are provided in Figure 1. The means (SD) of the test score distributions with 20, 40, and 80 items were 10.35 (4.52), 21.40 (8.43), and 44.03 (16.06) for test *X* and 7.58 (4.24), 18.17 (8.22), and 35.95 (16.89) for test *Y*.

In order to systematically verify the accuracy of the modified method of calculating the SEE as well as to explore how well it performs in a variety of sample sizes, sample sizes 1000, 4000, and 16,000 were considered. The study was conducted using version 3.6.2 of the statistical software environment R (R Core Team, 2019), primarily employing the packages *kequate* (Andersson et al., 2013), *mirt* (Chalmers, 2012), and *numDeriv* (Gilbert & Varadhan, 2019), while also utilizing newly written code implementing the modified SEEs (available in the Supplementary material). In each simulation setting, we used 10,000 replications which enabled us to all but eliminate the simulation random error. The convergence rate for all simulation settings was 100%.

The study followed the recommended kernel equating procedure (von Davier et al., 2004), albeit with a few adjustments to verify the derivations presented in this article. For each generated data set, the following steps were carried out:

*(1) Pre-smoothing.* The package *mirt* (Chalmers, 2012) was used to pre-smooth the irregularities of the raw data by estimating two separate 2-PL models to obtain item parameter estimates pertaining to each of the two groups and tests. The expectation-maximization (EM) algorithm was used for estimation (Bock & Aitkin, 1981), with the tolerance level 0.0001 and maximum number of iterations equal to 500. The asymptotic covariance matrix was estimated based on the method described in Oakes (1999).

*(2) Estimation of score probabilities.* Under the equivalent groups design, the score probabilities ${\hat{r}}_{j}$ and ${\hat{s}}_{k}$ were estimated based on the item parameter estimates and the assumed distribution of the latent variable (Andersson & Wiberg, 2017; von Davier et al., 2004).

*(3) Continuous approximation.* By adapting code from the package *kequate* (Andersson et al., 2013), continuous approximations to the discrete distributions were obtained by applying a Gaussian kernel with an optimal bandwidth parameter. Optimal bandwidth parameters ${\hat{h}}_{X}$ and ${\hat{h}}_{Y}$ were obtained by minimizing the first part of the penalty function, PEN_1_ (von Davier et al., 2004). When optimizing the penalty function, a golden section search with successive parabolic interpolation (Brent, 1973) using the default tolerance of 1.50 × 10^−8^ was used.

The analytical derivations for the bandwidth parameter estimator variance were paramount to the study. Hence, upon obtaining the optimal bandwidths, the average standard errors of the bandwidth parameters were computed following the equations introduced in the previous section, and their accuracy was assessed using the Monte Carlo standard error (MCSE) as the criterion. When calculating the asymptotic variance of the bandwidth parameter estimator, the bandwidth parameters *h*_*X*_ and *h*_*Y*_ were replaced with the estimated parameters ${\hat{h}}_{X}$ and ${\hat{h}}_{Y}$ , and the asymptotic covariance matrices of the estimated score probabilities Σr^ and Σs^ were calculated based on the implementation in *kequate* (Andersson et al., 2013).

*(4) Equating*. Upon obtaining continuous CDFs, an equipercentile equating function was applied to equate the test forms *X* and *Y*.

*(5) Calculating the SEE.* The average analytical SEEs were computed using the original method for calculating the SEE without accounting for the bandwidth variability (Holland et al., 1989), and the modified method of calculating the SEE accounting for the bandwidth variability. The Monte Carlo SEEs (MCSEE) were used as a criterion for comparing the accuracy of the modified and the original methods of the SEE calculation.

Furthermore, two measures were used to assess the performance and the accuracy of the modified method as compared to the original method. We computed the absolute differences of the means of the SEEs calculated with the original and the modified methods. Additionally, the average coverage probabilities were considered which explored the average proportion of time that the 95% confidence intervals calculated employing the original and the modified methods contained the true values of the equated results. The confidence intervals were estimated with ${\hat{e}}_{Y}\pm z_{0.975}\times \hat{\text{SE}}\left({\hat{e}}_{Y}\right)$ , with *z*_0.975_ indicating the 0.975 quantile of the standard normal distribution.

The analytical derivations used in computing the bandwidth estimator variance and standard errors, as well as the SEE, were verified numerically using the R package *numDeriv* (Gilbert & Varadhan, 2019). The R code is available for review in the Supplementary material.

### Simulation Results

The study largely depended on the accuracy of the asymptotic variance and standard error of the bandwidth parameter estimator derivations. The results of the simulation for the standard errors of the bandwidth parameter estimators ${\hat{h}}_{X}$ and ${\hat{h}}_{Y}$ given in Table 1 confirmed that the derivations were correct, and the asymptotic standard errors of the bandwidth parameter estimator (ASE) were accurate as witnessed by the comparison to the MCSE. As can be expected for asymptotic variance approximation (Ferguson, 1996), the differences between the ASEs and the MCSEs were larger in smaller sample sizes.

**Table 1.** Asymptotic Standard Errors (ASE) and Monte Carlo Standard Errors (MCSE) for the Bandwidth Parameters *h*_*X*_ and *h*_*Y*_ with Sample Sizes *N* and Test Lengths of 20, 40, and 80 Items.

	*h*_*X*_ Parameter		*h*_*Y*_ Parameter	
N	ASE	MCSE	ASE	MCSE
*20 items*				
1000	0.0035	0.0035	0.0035	0.0035
4000	0.0018	0.0017	0.0018	0.0017
16000	0.0009	0.0009	0.0009	0.0009
*40 items*				
1000	0.0034	0.0034	0.0037	0.0037
4000	0.0017	0.0017	0.0019	0.0019
16000	0.0009	0.0009	0.0009	0.0009
*80 items*				
1000	0.0033	0.0033	0.0036	0.0036
4000	0.0016	0.0016	0.0018	0.0018
16000	0.0008	0.0008	0.0009	0.0009

Subsequently incorporating the bandwidth estimation variability into computing the modified SEEs, Table 2 presents two performance measures used to compare the accuracy of the original standard errors of equating (ASEE) and the modified asymptotic standard errors of equating (ASEE_mod_). These measures are the absolute aggregate differences between the SEEs for two pairs, ASEE - MCSEE and ASEE_mod_ - MCSEE, and the average coverage for both the original and the modified methods. From the average differences, it was evident that when compared to the MCSEE estimates, the modified asymptotic SEEs which take bandwidth variability into account were accurate for all sample sizes and test lengths. Furthermore, the modified asymptotic SEEs in most cases appeared to be nearly identical to those not accounting for bandwidth variability, suggesting that the bandwidth estimation influence on the SEEs was minimal. This finding was further supported by the average coverage for both the original and the modified methods. Although the modified method performed better in most settings, the differences in coverage were small.

**Table 2.** Absolute Average Differences for the Original Asymptotic Standard Errors of Equating (ASEE) and the Modified Asymptotic Standard Errors of Equating (ASEE_mod_) to the Monte Carlo Standard Errors of Equating (MCSEE) and Average Coverage of 95% Confidence Intervals Based on the ASEE and the ASEE_mod_.

	Average Differences		Average Coverage	
N	ASEE-MCSEE	ASEE_mod_-MCSEE	ASEE	ASEE_mod_
*20 items*				
1000	0.0012	0.0016	94.97	95.03
4000	0.0002	0.0004	95.07	95.13
16000	0.0004	0.0004	94.87	94.93
*40 items*				
1000	0.0021	0.0019	94.97	94.99
4000	0.0012	0.0011	94.82	94.84
16000	0.0011	0.0011	94.66	94.69
*80 items*				
1000	0.0038	0.0041	94.87	94.90
4000	0.0050	0.0054	95.39	95.42
16000	0.0004	0.0006	94.90	94.93

## Discussion

The kernel method of equating is an equipercentile equating method in which number-correct scores are transformed into percentile rank scores from test form *X* to the scale of test form *Y*, and the scores from the two test forms with the same percentile rank are considered to be equivalent (von Davier et al., 2004). However, in order to obtain those equivalent scores, continuous approximations to the discrete score distributions are needed. To satisfy this requirement, kernel equating uses a Gaussian kernel with a smoothing bandwidth parameter that determines the characteristics of the continuous approximations to the raw discrete distributions (von Davier et al., 2004). The most commonly used method for bandwidth estimation is minimizing a penalty function with respect to the bandwidth parameter (von Davier et al., 2004). The bandwidth, in turn, is influenced by the estimated score probabilities and therefore is subject to variability. This variability, however, is not currently accounted for when calculating the SEE (Holland et al., 1989), challenging its accuracy and, ultimately, the fairness of the equated results.

The present study explored the issue of the additional variability stemming from the bandwidth estimation and its impact on the SEE. Building on the existing methodology of Holland et al. (1989) and von Davier et al. (2004), we derived the asymptotic variance of the bandwidth parameter estimator using the delta method for implicit functions (Benichou & Gail, 1989) and incorporated those derivations to expand the existing formulas for calculating the SEE (Holland et al., 1989). Thus, we have introduced SEEs that account for bandwidth estimation variability. A simulation study with 18 data sets generated for a wide range of sample sizes and test lengths was used to illustrate the results of the modified method as compared to the current method of the SEE calculation (Holland et al., 1989) and the MCSEEs.

The results offered several observations which are valuable to the testing industry. Firstly, the newly introduced SEE were accurate and close to the MCSEE estimates for all sample sizes and test lengths, suggesting that the method is suitable for practical use. Secondly, using the MCSEE as a criterion, the results of the study indicate that the original (Holland et al., 1989) and the modified SEEs produce similar results, suggesting that the bandwidth estimation impact on the SEE is minimal.

The presented results apply directly to any pre-smoothing method, provided that the asymptotic covariance matrix of the score probabilities has been defined for such a method. However, in this study we only utilized IRT as the pre-smoothing method and the results may be different if instead using, for example, log-linear models. However, previous research has indicated that the SEEs are fairly accurate even when not accounting for the bandwidth estimation with the penalty function, and so we do not anticipate that the results will differ substantially when using pre-smoothing with log-linear models instead of IRT models.

The method for accounting for bandwidth estimation that we used in the present study can be generalized to additional kernels and equating designs by modifying the presented results to account for the different expressions of the equating function and score probabilities with such approaches. It is furthermore possible to utilize the delta method for implicit functions with other bandwidth estimation methods provided that those specify a function that is minimized which fulfills the properties required for the implicit function theorem and the delta method. One approach which does not fulfill these requirements is the method based on PEN_2_, since the function PEN_2_ is not differentiable and can have multiple local minima.

It is important to note that in this study, we derived the modified asymptotic SEE for two test forms in the setting of the equivalent groups data collection design. It can be the case that the bandwidth estimation influence on the SEE is greater for other data collection and equating designs. It would, therefore, be beneficial for future theoretical and empirical studies to focus on determining the bandwidth estimation impact on the SEE in these additional scenarios.

As a final note, we believe that it is theoretically more sound to use a method which successfully accounts for all sources of variability, however negligible those may be. Introducing the modifications to the formulas for the SEE calculation akin to those explored in this study can improve the accuracy of the standard errors of equating, and consequently, facilitate fairness and comparability of the equated results.

## Appendix Computation of the Penalty Function

In order to compute Equation 26, two partial derivatives of the PEN_1_ (*h*_*X*_) function need to be defined, the second-order partial derivative of PEN_1_ (*h*_*X*_) with respect to *h*_*X*_ and the second-order partial derivative of PEN_1_ (*h*_*X*_) with respect to *h*_*X*_ and ***r***. Hence, we first need to compute the partial derivative of PEN_1_ (*h*_*X*_) with respect to *h*_*X*_.

Recalling equations (8)–(13), we define the first partial derivative of PEN_1_ (*h*_*X*_) with respect to *h*_*X*_ as

(36)
$$\begin{array}{ll} \frac{\partial {\text{PEN}}_{1}}{\partial h_{X}} & =\frac{\partial }{\partial h_{X}}\left[\sum_{j}(r_{j}-f_{h_{X}}{\left(x_{j}\right)}^{2}\right]\\ & =-2\sum_{j}\left(\left(r_{j}-f_{h_{X}}\left(x_{j}\right)\right)\frac{\partial f_{h_{X}}\left(x_{j}\right)}{\partial h_{X}}\right). \end{array}$$

We then need to calculate $\frac{\partial f_{h_{X}}\left(x_{j}\right)}{\partial h_{X}}$ as

(37)
$$\begin{array}{ll} \frac{\partial f_{h_{X}}\left(x_{j}\right)}{\partial h_{X}} & =\frac{\partial }{\partial h_{X}}\left[\sum_{j}r_{j}\phi \left(R_{jX}\left(x\right)\right)\frac{1}{a_{X}h_{X}}\right]\\ & =\sum_{j}r_{j}\left(\frac{\partial \left[\phi \left(R_{jX}\left(x\right)\right)\right]}{\partial h_{X}}\frac{1}{a_{X}h_{X}}+\phi \left(R_{jX}\left(x\right)\right)\frac{\partial }{\partial h_{X}}\left[\frac{1}{a_{X}h_{X}}\right]\right), \end{array}$$

where $\frac{\partial \left[\phi \left(R_{jX}\left(x\right)\right)\right]}{\partial h_{X}}$ is

(38)
$$\frac{\partial \left[\phi \left(R_{jX}\left(x\right)\right)\right]}{\partial h_{X}}=-\phi \left(R_{jX}\left(x\right)\right)\frac{\partial R_{jX}\left(x\right)}{\partial h_{X}}R_{jX}\left(x\right),$$

with

(39)
$$\begin{array}{ll} \frac{\partial R_{jX}\left(x\right)}{\partial h_{X}} & =\frac{\partial }{\partial h_{X}}\left[\frac{x-a_{X}x_{j}-\left(1-a_{X}\right)\mu_{X}}{a_{X}h_{X}}\right]\\ & =\frac{\partial a_{X}}{\partial h_{X}}\left(\mu_{X}-x_{j}\right)\frac{1}{a_{X}h_{X}}+\left(x-a_{X}x_{j}-\left(1-a_{X}\right)\mu_{X}\right)\frac{\partial }{\partial h_{X}}\left[\frac{1}{a_{X}h_{X}}\right]. \end{array}$$

The remaining components needed for computing the first partial derivative of PEN_1_ (*h*_*X*_) with respect to *h*_*X*_ are then $\frac{\partial }{\partial h_{X}}\left[\frac{1}{a_{X}h_{X}}\right]$ and $\frac{\partial a_{X}}{\partial h_{X}}$ . Thus we calculate

(40)
$$\frac{\partial }{\partial h_{X}}\left[\frac{1}{a_{X}h_{X}}\right]=-a_{X}^{-2}\frac{\partial a_{X}}{\partial h_{X}}\frac{1}{h_{X}}-\frac{1}{a_{X}h_{X}^{2}},$$

(41)
$$\begin{array}{ll} \frac{\partial a_{X}}{\partial h_{X}} & =\frac{\partial }{\partial h_{X}}\left[\frac{\sigma_{X}}{\sqrt{\sigma_{X}^{2}+h_{X}^{2}}}\right]=-\frac{\sigma_{X}}{2{\left(h_{X}^{2}+\sigma_{X}^{2}\right)}^{\frac{3}{2}}}\frac{\partial h_{X}^{2}}{\partial h_{X}}+\frac{\partial \sigma_{X}^{2}}{\partial h_{X}}\\ & =-\frac{\sigma_{X}h_{X}}{{\left(h_{X}^{2}+\sigma_{X}^{2}\right)}^{\frac{3}{2}}}. \end{array}$$

Using equations (36)–(41), we can compute the second partial derivative of PEN_1_ (*h*_*X*_) with respect to *h*_*X*_ as

(42)
$$\begin{array}{ll} \frac{\partial^{2}{\text{PEN}}_{1}}{\partial h_{X}^{2}} & =\frac{\partial }{\partial h_{X}}\left[-2\sum_{j}\left(\left(r_{j}-f_{h_{X}}\left(x_{j}\right)\right)\frac{\partial f_{h_{X}}\left(x_{j}\right)}{\partial h_{X}}\right)\right]\\ & =-2\sum_{j}\left(\frac{\partial^{2}f_{h_{X}}\left(x_{j}\right)}{\partial h_{X}^{2}}\left(r_{j}-f_{h_{X}}\left(x_{j}\right)\right)-{\left[\frac{\partial f_{h_{X}}\left(x_{j}\right)}{\partial h_{X}}\right]}^{2}\right), \end{array}$$

where $\frac{\partial f_{h_{X}}\left(x_{j}\right)}{\partial h_{X}}$ is defined in Equation 37 and $\frac{\partial^{2}f_{h_{X}}\left(x_{j}\right)}{\partial h_{X}^{2}}$ is given by

(43)
$$\begin{array}{ll} \frac{\partial^{2}f_{h_{X}}\left(x_{j}\right)}{\partial h_{X}^{2}} & =\frac{\partial }{\partial h_{X}}\left[\sum_{j}r_{j}\left(\frac{\partial [\phi \left(R_{jX}\left(x\right)\right)]}{\partial h_{X}}\frac{1}{a_{X}h_{X}}+\phi \left(R_{jX}\left(x\right)\right)\frac{\partial }{\partial h_{X}}\left[\frac{1}{a_{X}h_{X}}\right]\right)\right]\\ & =\sum_{j}r_{j}\left(\frac{\partial^{2}[\phi \left(R_{jX}\left(x\right)\right)]}{\partial h_{X}^{2}}\frac{1}{a_{X}h_{X}}+\frac{\partial [\phi \left(R_{jX}\left(x\right)\right)]}{\partial h_{X}}\frac{\partial }{\partial h_{X}}\left[\frac{1}{a_{X}h_{X}}\right]\right)\\ & \;+\sum_{j}r_{j}\left(\frac{\partial [\phi \left(R_{jX}\left(x\right)\right)]}{\partial h_{X}}\frac{\partial }{\partial h_{X}}\left[\frac{1}{a_{X}h_{X}}\right]+\phi \left(R_{jX}\left(x\right)\right)\frac{\partial^{2}}{\partial h_{X}^{2}}\left[\frac{1}{a_{X}h_{X}}\right]\right). \end{array}$$

Recall that $\frac{\partial \left[\phi \left(R_{jX}\left(x\right)\right)\right]}{\partial h_{X}}$ is given in Equation 38 and $\frac{\partial R_{jX}\left(x\right)}{\partial h_{X}}$ in Equation 39. Hence, we define $\frac{\partial^{2}\left[\phi \left(R_{jX}\left(x\right)\right)\right]}{\partial h_{X}^{2}}$ and $\frac{\partial^{2}R_{jX}\left(x\right)}{\partial h_{X}^{2}}$ as

(44)
$$\begin{array}{ll} \frac{\partial^{2}[\phi \left(R_{jX}\left(x\right)\right)]}{\partial h_{X}^{2}} & =\frac{\partial }{\partial h_{X}}\left[-\phi \left(R_{jX}\left(x\right)\right)\frac{\partial R_{jX}\left(x\right)}{\partial h_{X}}R_{jX}\left(x\right)\right]\\ & =-\frac{\partial [\phi \left(R_{jX}\left(x\right)\right)]}{\partial h_{X}}\frac{\partial R_{jX}\left(x\right)}{\partial h_{X}}R_{jX}\left(x\right)-\phi \left(R_{jX}\left(x\right)\right)\frac{\partial^{2}R_{jX}\left(x\right)}{\partial h_{X}^{2}}R_{jX}\left(x\right)\\ & -\phi \left(R_{jX}\left(x\right)\right){\left[\frac{\partial [R_{jX}\left(x\right)]}{\partial h_{X}})\right]}^{2}, \end{array}$$

(45)
$$\begin{array}{ll} \frac{\partial^{2}R_{jX}\left(x\right)}{\partial h_{X}^{2}} & =\frac{\partial }{\partial h_{X}}\left[\frac{\partial a_{X}}{\partial h_{X}}\left(\mu_{X}-x_{j}\right)\frac{1}{a_{X}h_{X}}+\left(x-a_{X}x_{j}-\left(1-a_{X}\right)\mu_{X}\right)\frac{\partial }{\partial h_{X}}\left[\frac{1}{a_{X}h_{X}}\right]\right]\\ & =\left(\mu_{X}-x_{j}\right)\left(\frac{\partial^{2}a_{X}}{\partial h_{X}^{2}}\frac{1}{a_{X}h_{X}}+\frac{\partial a_{X}}{\partial h_{X}}\frac{\partial }{\partial h_{X}}\left[\frac{1}{a_{X}h_{X}}\right]\right)\\ & \;\;\;+\left(\mu_{X}-x_{j}\right)\frac{\partial a_{X}}{\partial h_{X}}\frac{\partial }{\partial h_{X}}\left[\frac{1}{a_{X}h_{X}}\right]+\left(x-a_{X}x_{j}-\left(1-a_{X}\right)\mu_{X}\right)\frac{\partial^{2}}{\partial h_{X}^{2}}\left[\frac{1}{a_{X}h_{X}}\right]. \end{array}$$

Consider further that $\frac{\partial }{\partial h_{X}}\left[\frac{1}{a_{X}h_{X}}\right]$ is defined in Equation 40 and $\frac{\partial a_{X}}{\partial h_{X}}$ in Equation 41. We can then observe that $\frac{\partial^{2}}{\partial h_{X}^{2}}\left[\frac{1}{a_{X}h_{X}}\right]$ can be computed as

(46)
$$\begin{array}{ll} \frac{\partial^{2}}{\partial h_{X}^{2}}\left[\frac{1}{a_{X}h_{X}}\right]= & \frac{1}{h_{X}a_{X}^{2}}\left(\frac{2}{a_{X}}{\left[\frac{\partial a_{X}}{\partial h_{X}}\right]}^{2}-\frac{\partial^{2}a_{X}}{\partial h_{X}^{2}}+\frac{\partial a_{X}}{\partial h_{X}}\frac{1}{h_{X}}\right)\\ & +\left(\frac{\partial a_{X}}{\partial h_{X}}\frac{1}{a_{X}^{2}h_{X}^{2}}+\frac{2}{a_{X}h_{X}^{3}}\right), \end{array}$$

and

(47)
$$\frac{\partial^{2}a_{X}}{\partial h_{X}^{2}}=\frac{\partial }{\partial h_{X}}\left[-\frac{\sigma_{X}h_{X}}{{\left(h_{X}^{2}+\sigma_{X}^{2}\right)}^{\frac{3}{2}}}\right]=\frac{-\sigma_{X}}{{\left(h_{X}^{2}+\sigma_{X}^{2}\right)}^{\frac{3}{2}}}-\frac{3h_{X}^{2}}{{\left(h_{X}^{2}+\sigma_{X}^{2}\right)}^{\frac{5}{2}}}.$$

Lastly, we can compute the second partial derivative of PEN_1_ (*h*_*X*_) with respect to ***r*** as follows

(48)
$$\begin{array}{ll} \frac{\partial^{2}{\text{PEN}}_{1}}{\partial h_{X}\partial r_{i}} & =\frac{\partial }{\partial r_{i}}\left[-2\sum_{j}\left(\left(r_{j}-f_{h_{X}}\left(x_{j}\right)\right)\frac{\partial f_{h_{X}}(x_{j})}{\partial h_{X}}\right)\right]\\ & =-2\sum_{j}\left[\left(\frac{\partial r_{j}}{\partial h_{X}\partial r_{i}}-\frac{\partial f_{h_{X}}(x_{j})}{\partial r_{i}}\right)\frac{\partial f_{h_{X}}(x_{j})}{\partial h_{X}}+\left(r_{j}-f_{h_{X}}\left(x_{j}\right)\right)\frac{\partial^{2}f_{h_{X}}(x_{j})}{\partial h_{X}\partial r_{i}}\right], \end{array}$$

where $\frac{\partial r_{j}}{\partial h_{X}\partial r_{i}}=1$ if *i* = *j* and $\frac{\partial r_{j}}{\partial h_{X}\partial r_{i}}=0$ if *i* ≠ *j*. Note that $\frac{\partial f_{h_{X}}\left(x_{j}\right)}{\partial h_{X}}$ is given in Equation 37. Then, the components needed for computing Equation 48 are $\frac{\partial f_{h_{X}}\left(x_{j}\right)}{\partial r_{i}}$ and $\frac{\partial^{2}f_{h_{X}}\left(x_{j}\right)}{\partial h_{X}\partial r_{i}}$ . We define $\frac{\partial f_{h_{X}}\left(x_{j}\right)}{\partial r_{i}}$ as

(49)
$$\begin{array}{ll} \frac{\partial f_{h_{X}}\left(x\right)}{\partial r_{i}} & =\frac{\partial }{\partial r_{i}}\left[\sum_{j}r_{j}\phi \left(R_{jX}\left(x\right)\right)\frac{1}{a_{X}h_{X}}\right]\\ & =\frac{1}{h_{X}}\left[\phi \left(R_{jX}\left(x\right)\right)\frac{1}{a_{X}}-\frac{\partial R_{jX}\left(x\right)}{\partial r_{i}}\sum_{j}\left(r_{j}\phi \left(R_{jX}\left(x\right)\right)R_{jX}\left(x\right)\right)\frac{1}{a_{X}}\right]\\ & \;\;\;+\frac{1}{h_{X}}\left[\frac{\partial }{\partial r_{i}}\left[\frac{1}{a_{X}}\right]\sum_{j}\left(r_{j}\phi \left(R_{jX}\left(x\right)\right)\right)\right], \end{array}$$

where $\frac{\partial R_{jX}}{\partial r_{i}}$ and $\frac{\partial }{\partial r}\left[\frac{1}{a_{X}}\right]$ are given in Holland et al. (1989) as

(50)
$$\frac{\partial }{\partial r_{i}}\left[\frac{1}{a_{X}}\right]=-\frac{1}{2}a_{X}\frac{h_{X}^{2}}{\sigma_{X}^{2}}\frac{x_{i}^{2}-\mu_{X}^{2}}{\sigma_{X}^{2}},$$

and

(51)
$$\frac{\partial R_{jX}}{\partial r_{i}}=\left(-\frac{1}{a_{X}h_{X}}\right)\left[\frac{1}{2}\left(x-\mu_{X}\right)\left(1-a_{X}^{2}\right)\left(\frac{x_{i}^{2}-\mu_{X}^{2}}{\sigma_{X}^{2}}\right)+\left(1-a_{X}\right)x_{i}\right].$$

We further define $\frac{\partial^{2}f_{h_{X}}\left(x_{j}\right)}{\partial h_{X}\partial r_{i}}$ as

(52)
$$\frac{\partial^{2}f_{h_{X}}\left(x_{j}\right)}{\partial h_{X}\partial r_{i}}=\frac{\partial }{\partial h_{X}}\left[\frac{\partial f_{h_{X}}\left(x_{j}\right)}{\partial r_{i}}\right].$$

Given Equation 49 is a lengthy expression, we further simplify the notation such that

(53)
$$\begin{array}{ll} \frac{\partial }{\partial h_{X}}\left[\frac{\partial f_{h_{X}}\left(x_{j}\right)}{\partial r_{i}}\right] & =\frac{\partial }{\partial h_{X}}\left[\frac{1}{h_{X}}\right]\times P+\frac{1}{h_{X}}\times \frac{\partial P}{\partial h_{X}}\\ & =-\frac{1}{h_{X}^{2}}\times P+\frac{1}{h_{X}}\times \frac{\partial P}{\partial h_{X}}, \end{array}$$

where

(54)
$$\begin{array}{ll} P= & \frac{\partial \left[\phi \left(R_{jX}\left(x\right)\right)\right]}{\partial h_{X}}\frac{1}{a_{X}}-\frac{\partial R_{jX}\left(x\right)}{\partial r_{i}}\sum_{j}\left(r_{j}\phi \left(R_{jX}\left(x\right)\right)R_{jX}\left(x\right)\right)\frac{1}{a_{X}}\\ & +\frac{\partial }{\partial r_{i}}\left[\frac{1}{a_{X}}\right]\sum_{j}\left(r_{j}\phi \left(R_{jX}\left(x\right)\right)\right). \end{array}$$

Noting the three components in Equation 54, $\frac{\partial P}{\partial h_{X}}$ can then be presented as follows

(55)
$$\frac{\partial P}{\partial h_{X}}=\frac{\partial P1}{\partial h_{X}}-\frac{\partial P2}{\partial h_{X}}+\frac{\partial P3}{\partial h_{X}}.$$

$\frac{\partial P1}{\partial h_{X}}$ is given by

(56)
$$\begin{array}{ll} \frac{\partial P1}{\partial h_{X}} & =\frac{\partial }{\partial h_{X}}\left[\phi \left(R_{jX}\left(x\right)\right)\frac{1}{a_{X}}\right]\\ & =\frac{\partial \left[\phi \left(R_{jX}\left(x\right)\right)\right]}{\partial h_{X}}\frac{1}{a_{X}}+\phi \left(R_{jX}\left(x\right)\right)\frac{a_{X}h_{X}}{\sigma_{X}^{2}}, \end{array}$$

where $\frac{\partial \left[\phi \left(R_{jX}\left(x\right)\right)\right]}{\partial h_{X}}$ is given in Equation 38. $\frac{\partial P2}{\partial h_{X}}$ is defined as

(57)
$$\begin{array}{ll} \frac{\partial P2}{\partial h_{X}} & =\frac{\partial }{\partial h_{X}}\left[\frac{\partial R_{jX}\left(x\right)}{\partial r_{i}}\sum_{j}\left(r_{j}\phi \left(R_{jX}\left(x\right)R_{jX}\left(x\right)\right)\right)\frac{1}{a_{X}}\right]\\ & =\frac{\partial^{2}R_{jX}\left(x\right)}{\partial h_{X}\partial r_{i}}\sum_{j}\left(r_{j}\phi \left(R_{jX}\left(x\right)\right)R_{jX}\left(x\right)\right)\frac{1}{a_{X}}\\ & \;\;\;\;+\frac{\partial R_{jX}\left(x\right)}{\partial r_{i}}\sum_{j}\left(r_{j}\frac{\partial \left[\phi \left(R_{jX}\left(x\right)\right)\right]}{\partial h_{X}}R_{jX}\left(x\right)+r_{j}\phi \left(R_{jX}\left(x\right)\right)\frac{\partial R_{jX}\left(x\right)}{\partial h_{X}}\right)\frac{1}{a_{X}}\\ & \;\;\;\;+\frac{\partial R_{jX}\left(x\right)}{\partial r_{i}}\sum_{j}\left(r_{j}\phi \left(R_{jX}\left(x\right)R_{jX}\left(x\right)\right)\right)\frac{a_{X}h_{X}}{\sigma_{X}^{2}}, \end{array}$$

where $\frac{\partial R_{jX}\left(x\right)}{\partial r_{i}}$ is defined in Equation 51, $\frac{\partial \left[\phi \left(R_{jX}\left(x\right)\right)\right]}{\partial h_{X}}$ in Equation 38, and $\frac{\partial^{2}R_{jX}\left(x\right)}{\partial h_{X}\partial r_{i}}$ is given by

(58)
$$\begin{array}{ll} \frac{\partial^{2}R_{jX}\left(x\right)}{\partial h_{X}\partial r_{i}}= & \frac{\partial }{\partial h_{X}}\left[\left(-\frac{1}{a_{X}h_{X}}\right)\left(\frac{1}{2}\left(x-\mu_{X}\right)\left(1-a_{X}^{2}\right)\left(\frac{x^{2}-\mu_{X}^{2}}{\sigma_{X}^{2}}\right)+\left(1-a_{X}\right)x\right)\right]\\ = & \left(\frac{1}{a_{X}h_{X}^{2}}-\frac{a_{X}}{\sigma_{X}^{2}}\right)\left(\frac{1}{2}\left(x-\mu_{X}\right)\left(1-a_{X}^{2}\right)\left(\frac{x_{i}^{2}-\mu_{X}^{2}}{\sigma_{X}^{2}}\right)+\left(1-a_{X}\right)x\right)\\ & +\left(-\frac{1}{a_{X}h_{X}}\right)\left(\left(-\left(x-\mu_{X}\right)\left(\frac{x^{2}-\mu_{X}^{2}}{\sigma_{X}^{2}}\right)a_{X}\frac{\partial a_{X}}{\partial h_{X}}\right)+x\frac{\partial a_{X}}{\partial h_{X}}\right). \end{array}$$

It remains to calculate $\frac{\partial P3}{\partial h_{X}}$ as follows

(59)
$$\begin{array}{ll} \frac{\partial P3}{\partial h_{X}} & =\frac{\partial }{\partial h_{X}}\left[\frac{\partial }{\partial r_{i}}\left[\frac{1}{a_{X}}\right]\sum_{j}\left(r_{j}\phi \left(R_{jX}\left(x\right)\right)\right)\right]\\ & =\frac{\partial^{2}}{\partial h_{X}\partial r_{i}}\left[\frac{1}{a}\right]\sum_{j}\left(r_{j}\phi \left(R_{jX}\left(x\right)\right)\right)+\frac{\partial }{\partial r_{i}}\left[\frac{1}{a_{X}}\right]\frac{\partial }{\partial h_{X}}\left[\sum_{j}\left(r_{j}\phi \left(R_{jX}\left(x\right)\right)\right)\right]\\ & =-\left(\left(\frac{x_{i}^{2}-\mu_{X}^{2}}{\sigma_{X}^{2}}\right)\frac{1}{2\sigma_{X}^{2}}\right)\left(\frac{\partial a_{X}}{\partial h_{X}}h_{X}^{2}+2a_{X}h_{X}\right)\sum_{j}\left(r_{j}\phi \left(R_{jX}\left(x\right)\right)\right)\\ & \;\;\;+\frac{\partial }{\partial r_{i}}\left[\frac{1}{a_{X}}\right]\sum_{j}\left(r_{j}\frac{\partial \left[\phi \left(R_{jX}\left(x\right)\right)\right]}{\partial h_{X}}\right), \end{array}$$

where $\frac{\partial }{\partial r_{i}}\left[\frac{1}{a_{X}}\right]$ is given in Equation 50, $\frac{\partial a_{X}}{\partial h_{X}}$ in Equation 41, and $\frac{\partial \left[\phi \left(R_{jX}\left(x\right)\right)\right]}{\partial h_{X}}$ in Equation 38.

The partial derivatives of the PEN_1_ (*h*_*Y*_) with respect to *h*_*Y*_, $\frac{\partial {\text{PEN}}_{1}}{\partial h_{\Upsilon }}$ , $\frac{\partial^{2}{\text{PEN}}_{1}}{\partial h_{\Upsilon }}$ and $\frac{\partial^{2}{\text{PEN}}_{1}}{\partial h_{\Upsilon }\partial s_{\iota }}$ , are computed analogously.
